# High frequency of horizontal transfer in Jockey families (LINE order) of drosophilids

**DOI:** 10.1186/s13100-019-0184-1

**Published:** 2019-11-04

**Authors:** Izabella L. Tambones, Annabelle Haudry, Maryanna C. Simão, Claudia M. A. Carareto

**Affiliations:** 1 Department of Biology, Institute of Biosciences, Humanities and Exact Sciences (IBILCE), UNESP – São Paulo State University, Campus São José do Rio Preto, São Paulo, SP 15054-000 Brazil; 2 Laboratoire de Biométrie et Biologie Evolutive, Université de Lyon, Université Lyon 1, CNRS, UMR 5558, F-69622 Villeurbanne, France

**Keywords:** transposable elements, HTT, *Drosophila*, *Zaprionus*, *Scaptodrosophila*, VHICA

## Abstract

**Background:**

The use of large-scale genomic analyses has resulted in an improvement of transposable element sampling and a significant increase in the number of reported HTT (horizontal transfer of transposable elements) events by expanding the sampling of transposable element sequences in general and of specific families of these elements in particular, which were previously poorly sampled. In this study, we investigated the occurrence of HTT events in a group of elements that, until recently, were uncommon among the HTT records in *Drosophila* – the Jockey elements, members of the LINE (long interspersed nuclear element) order of non-LTR (long terminal repeat) retrotransposons. The sequences of 111 Jockey families deposited in Repbase that met the criteria of the analysis were used to identify Jockey sequences in 48 genomes of Drosophilidae (genus *Drosophila*, subgenus *Sophophora*: melanogaster, obscura and willistoni groups; subgenus *Drosophila*: immigrans, melanica, repleta, robusta, virilis and grimshawi groups; subgenus *Dorsilopha*: busckii group; genus/subgenus *Zaprionus* and genus *Scaptodrosophila*).

**Results:**

Phylogenetic analyses revealed 72 Jockey families in 41 genomes. Combined analyses revealed 15 potential HTT events between species belonging to different genera and species groups of Drosophilidae, providing evidence for the flow of genetic material favoured by the spatio-temporal sharing of these species present in the Palaeartic or Afrotropical region.

**Conclusions:**

Our results provide phylogenetic, biogeographic and temporal evidence of horizontal transfers of the Jockey elements, increase the number of rare records of HTT in specific families of LINE elements, increase the number of known occurrences of these events, and enable a broad understanding of the evolutionary dynamics of these elements and the host species.

## Background

Transposable elements (TEs) are abundant mobile repetitive sequences in eukaryotes that are capable of moving from one place to another in the genome. The introduction of a TE into a *naïve* genome may occur by three mechanisms: (i) de novo, by means of recombination between pre-existing and degraded TE sequences that restores their coding capacity and transpositional activity; (ii) introgression of sequences consequent to reproduction between closely related species; and (iii) horizontal transfer (HT) of TE sequences between different species via a non-sexual route. After such introduction, the TEs can proliferate both throughout the host genome and throughout the population by vertical transfer (VT) from parents to offspring. During this phase, it is possible for the TE to be transferred horizontally to another genome or lost as a result of random processes or of purifying selection due to deleterious effects of the insertion (for a review, see [1–3]). TEs may escape extinction during vertical transmission across generations if HT occurs [4].

Horizontal transfer of TEs (HTT), a quite frequent phenomenon in prokaryotes, has been increasingly documented in eukaryotes. Among the events recorded in metazoans, most are reported in Drosophilidae, a Diptera family widely used to study TEs since their discovery as important sources of genetic variability and genomic evolution. Since the first report of HTT in the 1980s, i.e., the transfer of the *P* element from *Drosophila willistoni* to *D. melanogaster* [5, 6], the number of such studies in drosophilids has increased, doubling from 101 in 2008 (reviewed in [1, 2]) to 218 in 2010 [3], and 243 reports are currently documented [7, 8]. This increasing frequency illustrates how advances in comparative analyses of complete genomes improved the probability of HTT identification and revealed that the rate of HTT is variable between the different types of TEs. Of the 243 cases reported, 126 (51.9%) are of DNA transposons, 103 (42.4%) are of LTR (long terminal repeat) retrotransposons, and only 14 (5.8%) involve non-LTR retrotransposons. For non-LTR retrotransposons in particular, only six families of the LINE order are involved, such as *Jockey* (*D. melanogaster*/*D. funebris*), *F* (*D. melanogaster*/*D. yakuba*), *Doc* (*D. melanogaster*/*D. yakuba*), *I* (*D. simulans*/*D. melanogaster*) (reviewed in [1, 2, 8]; HTT-DB platform), and *Helena* and *BS* (*melanogaster* complex/subgenus *Zaprionus*) [9]. The same marked difference in the HTT rate of different types of TEs was also recently detected by Reiss et al. [10], in a study of 460 species covering 19 orders of arthropods that focused on one LTR retrotransposon (Copia), one non-LTR retrotransposon (Jockey) and one DNA transposon (Mariner). The authors found that the HTT rate of three widely distributed TE superfamilies in Arthropoda, namely, Mariner (DNA transposons), Copia (LTR retrotransposons) and Jockey (non-LTR retrotransposons), which differ in their transposition mode, was 52, 37 and 10%, respectively. Interestingly, in contrast to having a low HTT rate, the Jockey superfamily is the most diverse in terms of the number of families in each genome (~ 15 families on average per species versus 4 and 3.5 for Copia and Mariner, respectively) and species distribution (252 species versus 174 and 129 for Copia and Mariner, respectively). Also interesting is that although arthropods generally present a low rate of HTT, butterflies and moths show a large excess of HTT: 56 events in Lepidoptera compared with the expected average of 13.7. In contrast, only two events were registered in Drosophilidae, one involving *D. melanogaster* and the other in *D. kikkawai*, two species belonging to the melanogaster group of the genus *Drosophila*.

Jockey is a superfamily of non-LTR retrotransposons found only in Arthropoda. The full-length element is ~ 5 kb in size [11, 12] and is formed by two ORFs (open reading frames), the first with 568 aa residues and the second with 916 aa residues. In contrast to ORF1, ORF2, which encodes an apurinic endonuclease (APE) and a reverse transcriptase (RT), is well conserved, shows tree-like evolution [13] and is very appropriate for phylogenetic analyses. The low rate of HTT reported for this superfamily has been associated with the target-primed reverse transcription mode of transposition of the non-LTR elements, in which the cDNA strand is reverse-transcribed from an RNA template directly onto a chromosomal target site [14], thus not producing extra-chromosomal copies in the stable form of DNA.

The current study aimed to further investigate the rate of HTT in the Jockey superfamily, focusing on the occurrence of its families in drosophilids. For a definition of family, we followed Wicker et al. [15]: a family is a group of highly conserved TEs with a sequence similarity of ≥80% in at least 80% of their coding region in an aligned region of at least 80 nucleotides. The term clade (instead of superfamily) is used by the Repbase database [16], the most commonly used database for TE classification. Repbase also uses a somewhat different criterion from the TE hierarchical classification of Wicker et al. [15], based on studies of enzymology, structural similarities and relationships between sequences [17]. As an example, the 123 Jockey sequences within the *Jockey* clade are distributed in up to 15 families in each *Drosophila* species (named Jockey-1 to Jockey-15) in Repbase, and four Jockey families were recorded in *D. simulans* (Jockey-1_DSim, Jockey-2_DSim, Jockey-3_DSim, and Jockey-4_DSim). Because of the nomenclature criterion used by Repbase, the family Jockey-1_DSim has the same name (Jockey*-1*) but may not be closely related to the Jockey-1 family of other *Drosophila* species. Therefore, the criteria for family denomination used by Repbase does not allow identifying the sharing of families between species, either by HT or by VT.

It is important to highlight, as considered by Wicker et al. [15], that “the precise definition of a family is problematic because groups of TEs with similar features sometimes form a continuum of sequence homology; […] it seems that evolutionary lineages are sufficiently distinct to allow the borders of such a continuum to distinguish a family”. Because a family of TEs is a member of an evolutionary lineage, it is essential to consider that its sequences may not be restricted to a single species but shared by a group of species through VT or HTT and to use phylogenetic approaches to identify families. Given the paucity of HTT records for Jockey families in drosophilids (only three cases has been reported so far), one may wonder if the elements of this superfamily are less prone to HTT than elements from other families for which more HTT events have been documented [8]. This study aimed to broaden our understanding of the evolution of Jockey families in drosophilids and to provide an estimate of the rate of HTT involving these families by using phylogenetic approaches. To do so, we sampled 48 genomes of Drosophilidae for the occurrence of Jockey sequences by using the 111 families deposited in Repbase as a query.

## Results

A broad homology-based search using 111 downloaded consensus sequences of Jockey families deposited in the Repbase database allowed us to identify Jockey sequences in 44 genomes of Drosophilidae species (Additional file 1: Table S1) belonging to the genera *Drosophila* (subgenera *Sophophora*, *Drosophila*, and *Dorsilopha*), *Zaprionus* (subgenus *Zaprionus*) and *Scaptodrosophila* (Additional file 1: Table S2). Sequences not yet catalogued in Repbase (Additional file 1: Table S3) were annotated in 28 species. In the subgenus *Sophophora,* new Jockey sequences were annotated in 12 genomes: five in species in the melanogaster group (*D. biarmipes, D. mauritiana, D. malerkotliana, D. serrata,* and *D. suzukii*) and seven in the *obscura* group (*D. athabasca, D. guanche, D. lowei, D. miranda, D. obscura, D. persimilis,* and *D. pseudoobscura*). In the subgenus *Drosophila*, 11 genomes had new Jockey sequences: two in species of the *immigrans* group (*D. albomicans* and *D. nasuta*)*,* three in the *melanica* group (*D. melanica, D. micromelanica,* and *D. nigromelanica)*, one in the *repleta* group (*D. mojavensis*), three in the *virilis* group (*D. americana, D. montana,* and *D. novamexicana*) and two in the *robusta* group (*D. lacertosa* and *D. robusta*). In the *Dorsilopha* subgenus, only *D. busckii* presented Jockey sequences. In addition, three species of the *Zaprionus* genus (*Z. africanus, Z. gabonicus,* and *Z. indianus*) and one of the genus *Scaptodrosophila* (*S. lebanonensis*) presented Jockey sequences. Among these, the sequences of *D. malerkotliana*, *D. nasuta* and *D. robusta* were not used in the analyses due to their small sizes.

### Phylogenetic analysis of the jockey families

**Fig. 1:**
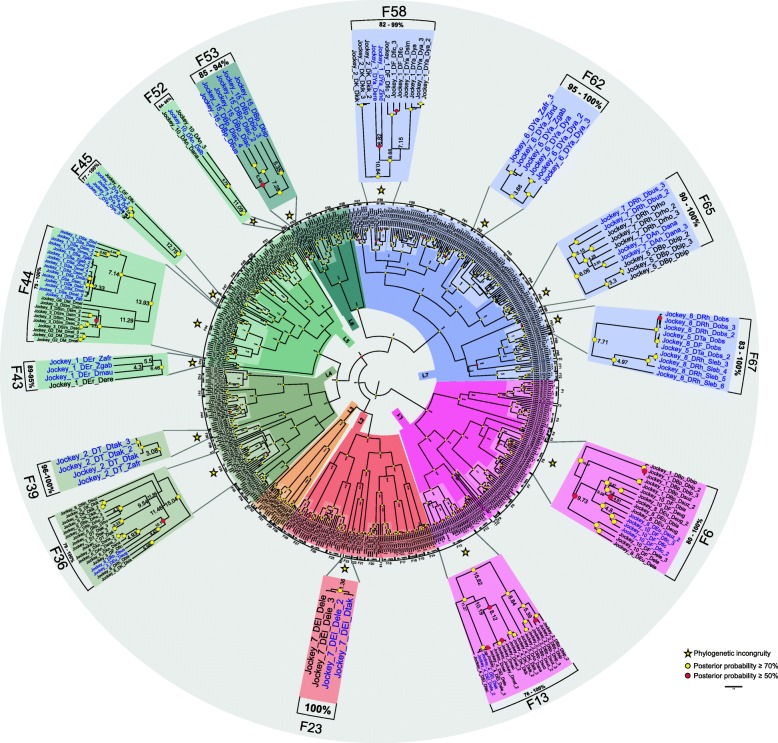
Phylogenetic relationships between sequences of Jockey families in drosophilids. The analysis involved 523 nucleotide sequences. The total data set included 446 positions. The evolutionary analyses were conducted in BEAST v16.1 [61]. The seven *Jockey* lineages are abbreviated by “Lin” and their respective number, and the 72 families are named “F”, followed by a number referring to the family number. Characterization of families was based on the p-distance, which was calculated with the pairwise deletion method for gap treatment in MEGA7 [63], and indicated in each bracket as similarity (%). Yellow circles: posterior probability ≥0.7, red circles: posterior probability ≥0.5%. In detail appear the 14 Jockey families in which phylogenetic incongruities (represented in blue) appear in relation to the species trees. Times of divergence from MRCA sequences calculated using BEAST v16.1 are given on the nodes

A phylogenetic tree was reconstructed using a multiple alignment of 523 RT sequences of Jockey families in 41 drosophilids that met the search criteria, in which the shortest sequence had 221 nt and the largest was 446 nt in length. To analyse the phylogeny, we considered the existence of Jockey evolutionary lineages and families. An evolutionary lineage is a large and robust monophyletic clade (posterior probability ≥70%) formed by several families. A family is constituted by Jockey sequences of the same or different species that share nucleotide similarity ≥80% and are grouped with ≥70% support (Fig. 1). In practical terms, two clades of the same lineage constitute different families when the minimum similarity between them is 80%. Exceptions to this criterion were allowed in three cases (families F13, F36 and F44), where a sequence was grouped with ≥75% similarity to a sequence group that constituted a family of the same evolutionary lineage if the average similarity of this family was ≥80. These criteria allowed us to classify all 523 *Drosophila* Jockey sequences into 72 families (F1 to F72), which were grouped into seven evolutionary lineages (Lin1 to Lin7). The number of families in each species varied from one (*D. athabasca*, *D. guanche*, *D. lowei*, *D. melanica*, *D. micromelanica*, *D. mojavensis*, *D. nigromelanica*, and *D. willistoni*) to 14 (*D. ananassae*) (Additional file 1: Table S3), indicating that the Jockey clade is very diverse in *Drosophila* genomes, as found in Arthropoda [10]. The divergence (p-distance) within (Additional file 2: Table S1) and between (Additional file 2: Table S2) the 72 Jockey families is given.

The most basal Jockey clade forms Lineage 1 (Fig. 1). This lineage is formed by 13 families, among which six families (F2, F4, F5, F9, F10 and F11) are formed by just one to three sequences of the same genome, which are divergent from the neighbouring clade (mean similarity: 62.5 to 75%). The other families include sequences of just one group or subgroup of species, such as the virilis group of the *Drosophila* subgenus (F1, F3, and F12) and the melanogaster subgroup of the *Sophophora* subgenus (F7). In addition, there are families formed by sequences of species belonging to different subgenera, such as the robusta group of the *Drosophila* subgenus and the obscura group of the *Sophophora* subgenus (F8), or to subgroups of the same species group (F6 and F13: subgroups of the melanogaster group). There is relative congruence between the phylogenetic relationships of the sequences within families F1, F3, F7, F8 and F12 and the species phylogeny (Fig. 1). However, F6 and F13 reveal incongruence with the species phylogeny. Family F6 includes two sequences of *D. suzukii*, one basal to the clade formed by *D. biarmipes*, *D. eugracilis*, *D. ficusphila* and *D. elegans* and the other closely related to sequences of *D. ficusphila* (average similarity = 96%), but not with the species of its own subgroup (*D. biarmipes*), representing the first phylogenetic incongruity in the tree. In addition, sequences of *D. elegans* and *D. takahashii* (F13) that belong to subgroups not closely related are clustered with a high degree of similarity (100%), representing the second incongruity.

Lineage 2 is also formed by 15 families (F14-F28), mostly represented by sequences of the obscura (F14, F15, F17, F20, F26 and F27) and virilis (F18, F25 and F28) groups, which follow the species phylogeny (Fig. 1). In this lineage, one sequence (Jockey_3_Dan_Dana) forms a single divergent family (F24), which clusters with F23 with support below 70%. Among the families formed by sequences from the melanogaster group (F21, F22, F23 and F24), the Oriental subgroups show the third phylogenetic incongruence. As also seen in F13, in F23 the sequences of *D. elegans* and *D. takahashii* are clustered together with high similarity (100%), which is not consistent with the phylogenetic relationship between the two sub-groups.. In turn, Lineage 3 is formed by only four families (F29-F32) that include only sequences of the melanogaster group (African and Oriental subgroups), three of them formed by sequences of a single genome (F30-F32).

Next, in the phylogenetic tree, 10 families (F33-F42) form Lineage 4 (Fig. 1), and four of them are constituted by sequences of a single genome (F33, F35, F37 and F41). The other families include sequences of the subgenus *Sophophora* (F34: obscura and F38, F40: Oriental melanogaster subgroups) and the subgenus *Drosophila* (F42: virilis group), which generally follows the species phylogeny. On the other hand, two incongruities are seen in this lineage. The clustering of sequences of *D. busckii* (busckii group) and *D. eugracilis* (melanogaster group) with high similarity (81%) in F36 reveals the fourth incongruity, considering that these sequences belong to species of two subgenera of the *Drosophila* genus, namely, *Dorsilopha* and *Sophophora*, respectively. The fifth incongruity can be seen in F39 because a sequence of *Z. indianus* (genus *Zaprionus*) clustered with sequences of *D. takahashii* (genus *Drosophila*), with average similarity incompatible with the phylogenetic relationships between the two groups of species (98%).

Ten families also compose Lineage 5 (F43-F52), of which four include the sequences of the melanogaster complex, all showing phylogenetic incongruities (Fig. 1). The sixth and seventh incongruities refer to *Zaprionus* species sequences: in F43, sequences of *Z. africanus* and *Z. gabonicus* cluster with a sequence of *D. mauritiana* (average similarity = 94%), and in F44, sequences of *Z. africanus*, *Z, gabonicus*, and *Z. indianus* cluster inside a clade with sequences of the melanogaster complex (average similarity = 95%). The eighth and ninth incongruities occur in F45 and F52, respectively, the first due to the clustering of a Jockey sequence of *D. simulans* inside a cluster of *D. yakuba* (average similarity = 98.4%) and the second involving two sequences of *D. ananassae* and a sequence of *D. albomicans* (immigrans group, *Drosophila* subgenus), which present a similarity equal to 86%. On the other hand, Lineage 6, which is composed of only five families (F53-F56), also presents an incongruity between species belonging to different subgenera in F53, the tenth incongruity of the phylogeny (Fig. 1), because the sequence of *D. busckii* is closely related (similarity = 85%) to sequences of *D. lacertosa* (robusta group, *Drosophila* subgenus).

The last lineage (Lin 7) is the largest (Fig. 1), composed of 15 Jockey families (F57-F72). The majority of Lin7 includes sequences of the melanogaster group (F57-F60, F63-F64, F68-F69 and F71-F72) or sequences clustered with those of other groups (F58, F62, F63 and F65), and sequences of the obscura group (F67) clustered with a sequence of *S. lebanonensis* and of *D. willistoni* (F70). Four incongruities can be observed in families that combine sequences of different species groups, subgenera or genera (F58, F62, F65 and F67). In the other families, the phylogenies of the Jockey sequences correspond relatively well to those of the species. The eleventh and twelfth incongruities involve sequences of *Zaprionus* clustered with sequences of species belonging to the melanogaster subgroup, the first with *D. erecta* (F58, similarity = 83%) and the second with *D. yakuba* (F62, similarity = 83%). The thirteenth and fourteenth incongruities occur in F65. The first is due to the clustering of *D. busckii* sequences with species of the Oriental melanogaster group with a similarity higher than expected (93%) between sequences belonging to species of two subgenera. The second is due to the clustering of *D. rhopaloa* and *D. ananassae* sequences, which belong to different subgroups (95%). Finally, in the fifteenth incongruity (F67), four sequences of *S. lebanonensis* cluster with sequences of *D. obscura* (similarity = 92%), thus involving two subgenera.

### Identification of jockey elements horizontal transfer

**Fig. 2:**
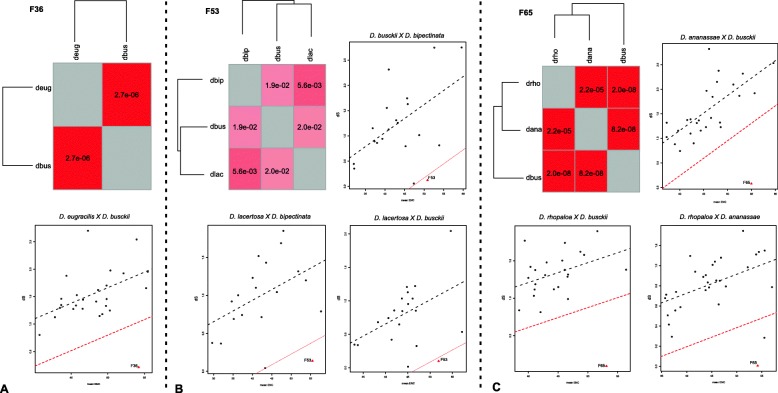
Identification of Jockey elements horizontal transfer. Consistency graphical representation and dS-ENC graphs obtained from the comparison between species in Jockey-F36 (**a**), Jockey-F53 (**b**) and Jockey-F65 (**c**) representing inferences of HTT. In the graphical matrix, the tree represents the host species and each square represents a pairwise comparison, which is coloured according to *p*-values calculated for the null hypothesis of VT: red represents significant signals of HTT, and the absence of colour represents VT (p-values indicated within each square). In the dS-ENC plots the black empty circles represent ENC–dS measures of the single-copy orthologous genes, the dotted black lines represent the linear regression of dS-ENC from genes, the dotted red lines represent the cut-off *P* value of 0.05 and red triangles represents the Jockey dS-ENC comparisons. dS: number of synonymous substitutions per synonymous site. ENC: effective number of codons. Species names are shortened as such: dbus for *D. busckii*, deug for *D. eugracilis*, dbip for *D. bipectinata,* dlac for *D. lacertosa*, dana for *D. ananassae* and drho for *D. rhopaloa*. There is evidence of HTT between all the species in the three Jockey families

Candidate cases of HTT were identified based on the 15 observed phylogenetic incongruities of Jockey elements (represented by stars in Fig. 1) from 14 families, hereafter named Jockey-F (1–72), belonging to six evolutionary strains. We used the VHICA (Vertical and Horizontal Inheritance Consistence Analysis) method based on comparison of dS rates of evolution and effective use of codons (ENC) to test for statistical support [18] of each inferred HTT event. Only the incongruities with a *p*-value < 0.01 were accepted as evidence for HTT: briefly, 15 phylogenetic incongruities were confirmed as HTTs. Among these, Fig. 2 shows the results of Jockey-F36, Jockey-F53 and Jockey-F65 obtained from dS-ENC pairwise comparisons between sequences of *D. busckii* and species of the Oriental melanogaster group with *D. eugracilis* (Jockey-F36), with *D. lacertosa*/*D. bipectinata* (Jockey-F53), and with *D. rophaloa*/*D. ananassae* (Jockey-F65), respectively. The linear regression between the *D. busckii* dS and ENC of Jockey sequences and the host genes is significant in F36 (Fig. 2a), as well as in F53 (Fig. 2b) and F65 (Fig. 2c), confirming the hypotheses of HTT based on phylogenetic incongruities. However, in Jockey-F53, all dS and ENC comparisons revealed significant differences, suggesting the occurrence of HTT involving the three species; hence, instead of one HTT, we should compute two, the first possibly between *D. bipectinata* and *D. lacertosa* and the second between this species and *D. busckii*. In addition, the comparison between the sequences of *D. rhopaloa* and *D. ananassae* in F65 also shows a significant signal of HTT. The positions of the points in the plots are beyond the limit of variance in the genes, supporting the hypothesis of five HTT events in these families. The consistency graph reflects these results, in which each square that represents a pairwise comparison is coloured according to the coloured bar of *p*-values calculated for the null hypothesis of VT.

**Fig. 3:**
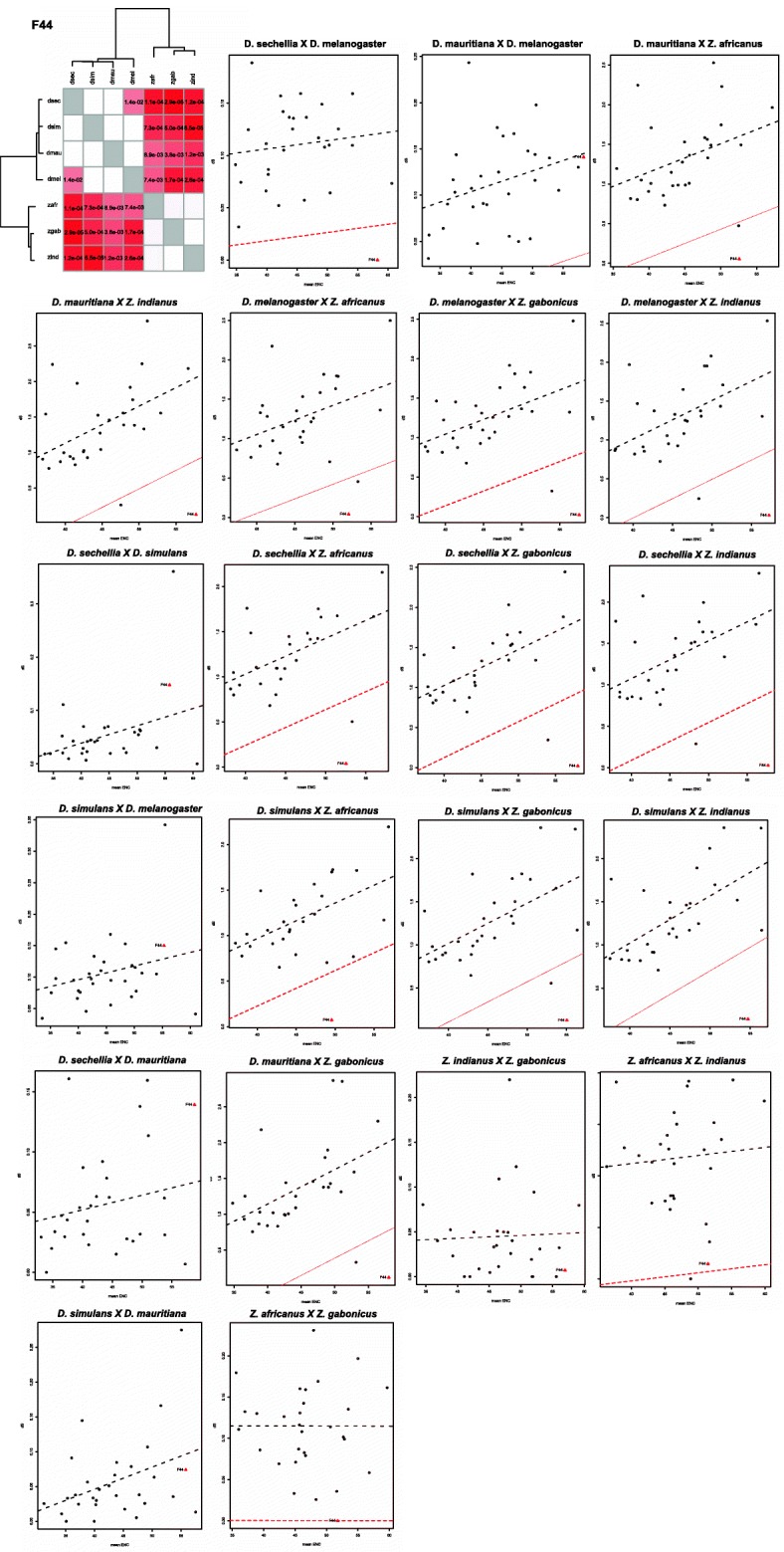
Identification of Jockey elements horizontal transfer. Consistency graphical representation and dS-ENC graphs obtained from the comparison between species in Jockey-F44 representing inferences of HTT and VT in species of the melanogaster complex and *Zaprionus*. Species names are shortened as such: dmel for *D. melanogaster*, dsech for *D. sechellia*, dsim for *D. simulans*, dmau for *D. mauritiana* and zafr for *Z. africanus*, zgab for *Z. gabonicus* and zind for *Z. indianus*. For details of the graphs refer to the legend of Fig. 2. There are signals of HTT between dmel and dsech, dmau and zafr/zgab/zind, dsech and zafr/zgab/zind and dsim and zafr/zgab/zind

**Fig. 4:**
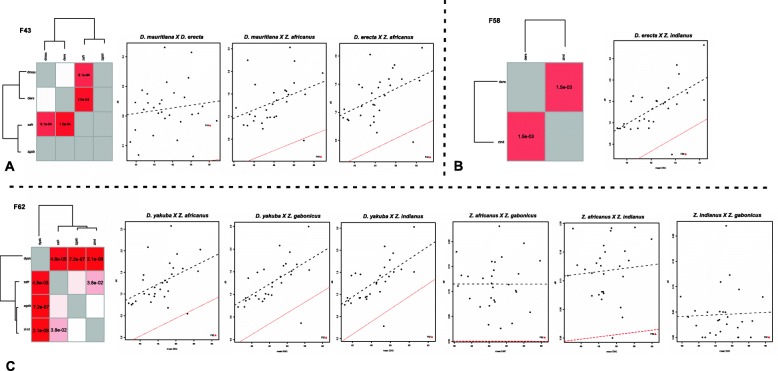
Identification of Jockey elements horizontal transfer. Consistency graphical representation and dS-ENC graphs obtained from the comparison between species in Jockey-F43 (**a**), Jockey-F58 (**b**) and Jockey-F62 (**c**) representing inferences of HTT and VT. Species names are shortened as such: dmau for *D. mauritiana*, dere for *D. erecta*, dyak for *D. yakuba* and zafr for *Z. africanus*, zgab for *Z. gabonicus* and zind for *Z. indianus*. For details of the graphs refer to the legend of Fig. 2. There are signals of HTT between zafr and dmau/dere (Jockey-F43), between dere and zind (Jockey-F58), dyak and zafr and zind

The results for the Jockey-F43-F44, F58 and F62 sequences were obtained from pairwise comparisons between species of the melanogaster subgroup and of the genus *Zaprionus*. The distribution of linear regressions between the dS and ENC values of Jockey-F44 sequences and of host genes of the species of the melanogaster complex (*D. melanogaster*, *D. simulans*, *D. sechellia* and D*. mauritiana*) and of the genus *Zaprionus* (*Z. africanus*, *Z. indianus* and *Z. gabonicus*) is beyond the limit of variance in the genes, supporting the hypothesis of HTT between these species (Fig. 3). In contrast, between the *Zaprionus* species and between the species of the melanogaster complex, there is no signal of HTT, with a sole exception: the position of Jockey-F44 in the graph is beyond the limit of variance in the comparison between *D. melanogaster* and *D. sechellia*. This result suggests the loss of Jockey-F44 ancestral copies in *D. sechellia* and reintroduction from *D. melanogaster*. Stochastic loss of mariner transposon has also been described in *D. sechellia*, as well as in other species of the melanogaster subgroup [19]. Similarly, the positions of the Jockey-F43 sequences of *Z. africanus* and both *D. erecta* and *D. mauritiana*, and *D. erecta* and *Z. indianus* (Fig. 4a), or also the *D. erecta* and *Z. indianus* sequences in F58 (Fig. 4b) are beyond the limit of variance in the genes, supporting the hypothesis of HTT between these species. A comparison between the Jockey-F43 sequences of *Z. gabonicus* with the others was not possible due to the length of the sequence, 189 bp, which resulted in only 63 codons. Additionally, significant signals of HTT are seen between the Jockey-F62 sequences of *D. yakuba* and the three *Zaprionus* species and between *Z. africanus* and *Z. indianus*, two sibling species [20] with very recent divergence (Fig. 4c). This signal of HTT is not supported by the significant difference criterion adopted here as well as could be a false-positive result because VHICA is not suitable for very closely related species [18], as well as due to incomplete lineage sorting; therefore, it was not considered HTT. The HTT signals between the three species of the genus *Zaprionus* and the species of the melanogaster subgroup, but not between the species within these groups, with the two exceptions aforementioned, suggest the occurrence of a single transfer involving ancestors of these species groups in the four Jockey families, and one transfer between *D. melanogaster* and *D. sechellia*, for a total of five HTTs.

**Fig. 5:**
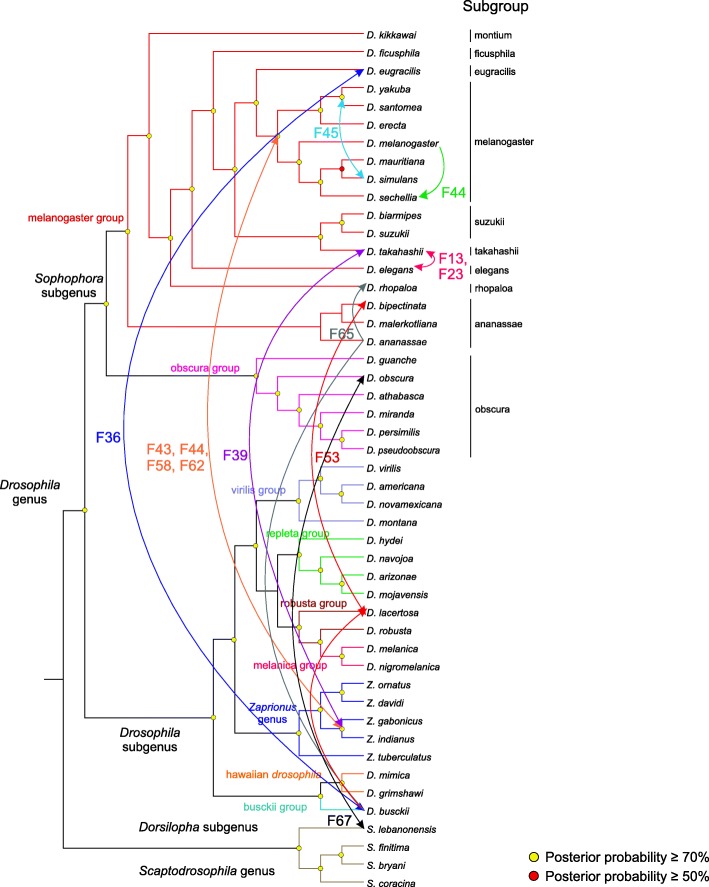
Phylogenetic relationships between sequences on the nuclear genes *Amyrel* in 48 species of drosophilids. Bayesian inference was performed with BEAST v16.1 [61] using General Time Reversible (plus Gamma distribution and invariable sites) as the substitution model. The evolutionary model of substitution that best fit the data was determined by the Find Best DNA Model [63]. The total data set included 1425 position. A posteriori phylogenetic support test was used, which involved the sampling of 100,000 trees with 10% burn-in. The arrows represent 15 HTT events between drosophilids of different genera (Jockey-F39, Jockey-F43, Jockey-F44, Jockey-F58, Jockey-F62, and Jockey-F67), between species groups of different subgenera (Jockey-F36, Jockey-F53 [2x] and Jockey-F65) and as well as between species of the same subgroup (Jockey-F13, Jockey-F23, Jockey-F45 and Jockey-F65) or complex (Jockey-F44)

Four incongruities occur between species of the melanogaster group, one among species belonging to the African subgroup, *D. simulans* and *D. yakuba* (Jockey-F45, Additional file 3: Figure S1), and three between species belonging to the Oriental subgroups (Jockey-F6, Jockey-F13 and Jockey-F23, Additional file 3: Figure S2). The linear regression between the *D. simulans-D. yakuba* dS and ENC is highly significant, supporting the occurrence of HTT. Although the phylogenetic relationships between the Oriental subgroups of the melanogaster group are to some extent uncertain [21], two incongruities reported above (Jockey-F13 and Jockey-F23) were validated as HTTs (Additional file 3: Figure S2 B and C). From the last three incongruities (Jockey-F39, Jockey-F52 and Jockey-F67), only two, namely, those between *D. takahashii* and *Z. africanus* (Additional file 3: Figure S3 A) and between *D. obscura* and *S. lebanonensis* (Additional file 3: Figure S3 B), were validated as HTTs, excluding that between *D. albomicans* and *D. ananassae* (Additional file 3: Figure S3C). The last two results should be interpreted with caution because only 12 (Jockey-F67) and 16 (Jockey-F52) host genes were obtained due to the poor genome quality of *S. lebanonensis* and *D. albomicans*. As pointed out by Wallau et al. [18], the use of few genes can lead to false positives and false negatives. In summary, in view of the premises established to propose a case of HTT, phylogenetic incongruence validated by significant discrepancies in the dS evolutionary rate between the Jockey elements and a set of vertically transferred genes of the host species, 15 phylogenetic incongruities were validated as potential cases of HTT. These transfers are depicted in Fig. 5.

## Discussion

The 41 genomes analysed here provided a broad sampling of Drosophilidae diversity, including three subgenera of the genus *Drosophila* (*Sophophora*, *Drosophila* and *Dorsilopha*) and two other genera, namely, *Zaprionus* and *Scaptodrosophila*. The genus *Scaptodrosophila* occupies a basal position within the Drosophilinae [22], and the placement of the genus *Zaprionus* within the subfamily stands for now, but there is a current consensus that it is imbedded within the genus *Drosophila*. The locations of origin of the species sampled are in all biogeographical regions where drosophilids occur, and several of the species are currently distributed worldwide.

To infer the occurrence of HTT, three main criteria should be considered: phylogenetic incongruence between the TE tree and the host tree; an irregular distribution of the element in a group of species; and high similarity between sequences of TEs from distantly related species [2, 23]. TEs are sets of copies dispersed throughout the genome of the host species, occurring in tens, hundreds or even thousands of families, which may have different levels of similarity between them and may or may not reflect common ancestry. Therefore, inferences of HTT are reliable if the TE phylogenetic relationships and classification are characterized with robustness; in such cases, we can be confident that the elements compared between two species belong to the same evolutionary unit (family).

### Identification and characterization of jockey families in Drosophilidae

The LINE elements of the Jockey superfamily are characterized by being exclusively present in arthropods, mainly insects [24]. The term family adopted here differs from that used by Repbase in that we consider a family of TEs to represent an evolutionary lineage that is often shared by several species. Accordingly, we propose that to infer events of HTT, sequences belonging to a family should be characterized following similarity (≥ 80%) and phylogenetic support (posterior probability ≥70%) criteria. Thus, the identification of sequences of a TE family in a species reflects the evolutionary relationships of each family, which may (VT) or may not (HTT) be congruent with the phylogenetic relationships of the host species. Following these criteria, we were able to characterize 72 Jockey families in 41 Drosophilidae species. It is important to emphasize that the robustness of HTT hypotheses in our study was initially based on the classical criteria for these kinds of inferences – a high sequence similarity and phylogenetic incongruities - associated with statistical support provided by the comparison of rates of evolution and codon usage among sequences [18]. However, HTT inferences become more reliable if they are based not only on the phylogenetic aspects but also on the biogeographic-evolutionary history of the species involved. The 15 candidate cases of HTT (20.8% of total families) suggested in this study are discussed in terms of this latter aspect plus the divergence times of the taxa (Fig. 1).

### The Orient: the point of origin and diversification of the melanogaster group

The melanogaster group, which originated in Asia approximately 50 Mya (million years ago), is one of the main groups of the genus *Drosophila* that inhabits the Old Continent [25]. All melanogaster subgroups diversified in the Oriental biogeographic region, except the melanogaster subgroup, which originated and diversified in Africa from a proto-melanogaster founder lineage that migrated to the African continent ~ 17 to 20 Mya [26–28]. In contrast to the melanogaster subgroup, the phylogenetic relationships and dating of divergence between the eastern subgroups are not fully elucidated [29–31], which causes difficulties in interpreting the phylogenetic incongruities between the Jockey sequences. In these cases, and in general inferences of HTT, the spatio-temporal overlap of species, which facilitated TE exchanges at some point in their evolutionary history, is an important criterion with which to add robustness to the inferences. Moreover, it is important to stress that unsampled species could also have been involved since species may share niches and direct or indirect interspecific ecological relations [32].

In the present study, we propose that due to sympatry, the possibility of sharing niches associated with the high movement of species during late Miocene may have contributed to the establishment of a scenario permissive to the occurrence of HTT involving four Jockey families in species of the Oriental melanogaster group. However, as the phylogenetic relationships between some Oriental melanogaster groups and their times of divergence are not completely established, the HTT inferences in these cases should be viewed with caution, even though they are supported by phylogenetic incongruities, later coalescence times of the sequence shared by two species than the species divergence times, and the significance of evolutionary rates and codon usage. Two of these proposed HTTs involve families that occur in species of the subgroups takahashii and elegans (Jockey-F13 and Jockey-F23). The Jockey most recent common ancestor (MRCA) sequence of the clade *D. takahashii/D. elegans* dates to only 1.58 Mya (F13) and 1.36 Mya (F23), times of divergence that are very recent for species belonging to different subgroups.

The Eastern and African groups of drosophilids, hosts of the Jockey families sampled here, may have participated in a large wildlife exchange between Eurasia and Africa, when an intercontinental route became possible for the first time between 17 and 20 Mya [26–28], or even more recently, between 6 and 13 Mya [33]. This high species dispersal may have facilitated genetic exchange among drosophilids, resulting in the high amount of HTT between several subgroups of the melanogaster group reported here for Jockey elements as well as elsewhere for other TEs (reviewed in [1, 2]). However, it is necessary to emphasize that not all phylogenetic incongruities are necessarily due to HTT because different evolutionary rates, ancestral polymorphism, stochastic loss, and introgressive hybridization also result in phylogenetic incongruities [2]. Moreover, incomplete lineage sorting (ILS) due to a short period of species divergence may also cause phylogenetic incongruities [34–36]. Phylogenomic studies of the 12 *Drosophila* genomes provide evidence of some ILS among *Drosophila* (30–40% of the loci) phylogenies, showing discrepancies with the species phylogeny [37]. The significant difference between dS and ENC of Jockey-F62 *Zaprionus* sequences and of host genes may be one of these cases. Another difficulty, in addition to the uncertainty about relationships between some groups of species, regards dating key nodes in the *Drosophila* phylogeny, which can vary widely, regardless of whether the calibration points used are from amber fossils [38], Hawaiian phylogeography [33, 39, 40] and island formation dates [41] or mutation rates [42]. In this study, we considered only HTT events as phylogenetic incongruities whose MRCA sequence dating was more recent than all the possible dates produced using these different calibration points.

### Tropical Africa: a permissive environment for HTT events

Tropical Africa was the location for the radiation of different groups of organisms, including drosophilids, mainly those belonging to the melanogaster subgroup and the *Zaprionus* subgenus. There is an important chapter in the history of the melanogaster group on the African continent, where the melanogaster subgroup originated from the proto-melanogaster lineage and gave rise to three speciation centres: the first, in the western region (15 to 13 Mya), produced the erecta complex (*D. erecta* and *D. orena*); the second, also originating in the western region (15 to 8 Mya), resulted in the yakuba complex (*D. yakuba*, *D. teissieri* and *D. santomea*); and the third gave rise to the melanogaster complex (*D. melanogaster* and the *simulans* subcomplex) approximately 3 to 2 Mya in central Africa. Later, an ancestral lineage of the simulans subcomplex (*D. simulans*, *D. sechellia* and *D. mauritiana*) dispersed to the islands of the Indian Ocean and Madagascar, approximately 400 thousand years ago (reviewed in 1). These periods are significantly more recent, based on dating using mutation rates as calibration points [41]. On the other hand, a lineage of the genus *Zaprionus* (Drosophilidae) also dispersed from Asia, reaching the African continent approximately 7 Mya [20] or even in the early Oligocene (~ 29.4 Mya, 29), giving rise to the African subgenus *Zaprionus*. The biogeographic history of this genus reveals possible geographic and temporal overlap with the species of the melanogaster subgroup. Thus, the species of the subgenus *Zaprionus*, have an evolutionary history distinct from that of the melanogaster group, and their biogeographic history reveals possible geographic and temporal overlap with the species of the melanogaster subgroup in the last 7 My.

The incongruities involving Jockey sequences of four families of two evolutionary lineages (Lin5: Jockey-F43 and Jockey-F44 and Lin7: Jockey-F58 and Jockey-F62), the high similarity, which reflects the recent times of divergence from the MRCA sequences, and the VHICA results strongly support the hypothesis of multiple HTT occurrences involving these families between species of the *Zaprionus* subgenus and of the melanogaster subgroup. The MRCAs of Jockey-F43sequences that *D. erecta* and *D. mauritiana* shares with *Z. gabonicus* and *Z. africanus* are dated to only 6.5 Mya or less Fig. 1). Jockey-F44 sequences of *Z. indianus*, *Z. gabonicus* and *Z. africanus* are imbedded within the clade containing sequences of the melanogaster complex, with the MRCA dating to 7.14 Mya or less. On the other hand, the MRCAs of the Jockey-F58 shared by *Z. indianus* and *D. erecta* and of Jockey-F62 shared by *Z. africanus*, *Z. indianus, Z. gabonicus* and *D. yakuba* are dated to 10.84 and 3.68 Mya, respectively. As we proposed earlier, the overlap in space and time during the radiation of the *Zaprionus* subgenus [20] and of the *melanogaster* subgroup in central Africa [26, 27] could have facilitated the exchange of Jockey sequences between ancestors of these two groups (Fig. 5).

Our research group has been dedicated to the study of HTT between *Zaprionus* and the species of the subgroup *melanogaster* of *Drosophila* for a decade. The results reported in this study for the Jockey families agree with the hypothesis of recent transfer of retrotransposons with LTR (Gypsy, Micropia and Copia) [42, 43] and the non-LTR retrotransposons *Helena* and *BS* [9] between genomes of species of the subgenus *Zaprionus* and the subgroup *melanogaster*. Thus, the evidence we add here provides a consolidated picture of the extensive exchange of TEs between the species of these two groups in central Africa during the late Miocene. In addition, there is evidence of HTT between *Z. africanus* and *D. takahashii* (Jockey-F39, Lin4). Since the MRCA of these sequences is dated to only 3.08 Mya, the hypothesis of HTT due to spatio-temporal overlap between the two groups of species cannot be applied if we assume the period of *Zaprionus* subgenus radiation in Africa (~ 7 Mya) given [20]. A possible explanation for this time incongruence is that a third species was involved in this transfer.

### Widespread species distribution and HTT

The rate of HTT depends on both the biology of TEs and various aspects of the species involved, such as their ecology, relatedness, and biology. From the TE point of view, studies indicate that DNA transposons move more often than LTR retrotransposons between species and more often than non-LTR retrotransposons due to the mode of transposition, which explains the low rate of HTT reported for Jockey (reviewed in [1–3, 18, 44]). Studies have also shown that HTT occurs more frequently between closely related species than between more distantly related ones [45–47]. Regarding the ecology and biology of the species involved in HTT, it has been shown that species with overlapping habitats share more TEs by HTT than species that are distributed in different environments [9, 42], as well as share more viruses that can be vectors of TEs [48]. Although susceptibility to viruses shows a strong phylogenetic correlation, susceptible hosts can occasionally be grouped into phylogenetically distant groups, allowing parasites to jump great phylogenetic distances [49].

Our data possibly show a further trend that can be added to the aforementioned ones - colonizing species that disperse across different geographic regions have increased HTT rates. It is noteworthy that of the 12 Jockey families in which HTT events were validated, in five (Jockey-F39, Jockey-F43, Jockey-F44, Jockey-F58, and Jockey-F62), the transfers occurred between two distantly related groups of species (the melanogaster group and *Zaprionus*) that migrated from the Eastern region to Africa. In addition, in three other families (Jockey-F36, Jockey-F53, and Jockey-F65), the transfer involved *D. busckii*, which is a cosmopolitan species [50] as well as *D. bipectinata*, which has wide distribution and spread across the Oriental-Australian biogeographic zone [51]. Additionally, in Jockey-F67, the HTT occurred between *D. obscura* and *S. lebanonensis*, which are widespread in the Palaeartic region. Moreover, although not validated by the VHICA and thus not considered an HTT event here, two extra incongruities involved widespread species, namely, *D. suzukii* [52] and *D. albomicans* [53], respectively in Jockey-F6 and Jockey-F52. The reason for the relationship between a widespread distribution and HTT needs to be investigated; however, what seems clear is that the ability of these species to disperse increases the chance of phylogenetic jumps of viruses, potential candidates to move TEs from one genome into another [54].

## Conclusion and perspectives

Recently, there has been an increase in the number of reported cases of HTT of non-LTR elements in *Drosophila*. Although only five cases were previously reported in drosophilids, involving only four LINE elements - Jockey, Doc, F and I [2], 20 cases were reported in Drosophilidae and 303 cases were reported in Insecta in 2017 [8] due to the use of large-scale genomic analyses [47, 55]. Despite this broad sampling, the identification of HTT events for specific families in the LINE order is lacking, and little is known about the species involved in these events. In the present study, we were able to characterize 15 new cases of HTT for Jockey families, which represents 20.8% of the families sampled in 41 species of *Drosophila*. This value is twice that obtained in Arthropoda, and this difference is due to poor sampling of drosophilids, with only nine species [10]. Equally importantly, we identified the species involved in these exchanges and formulated hypotheses about the spatio-temporal relationships between species that made these exchanges possible. While the HTT events between species of different subgenera were strongly corroborated, the inferences related to the eastern species of the *melanogaster* group, despite meeting all the criteria for HTT used in the literature, need greater support, which could be obtained by increasing the sampling of these elements in *Drosophila* species as well as producing more robust phylogenies of these species. Moreover, more complex phylogenetic approaches/models integrating ILS, duplication/loss and introgression should be developed.

## Material and methods

### Extraction of jockey sequences from genome assemblies

Genome assemblies for 48 species were downloaded from public databases (Additional file 1: Table S1): The European Nucleotide Archive (ENA, https://www.ebi.ac.uk/ena) for 31 of the 48, Ensembl Metazoa for the 12 first sequenced genomes [56], http://popoolation.at/ for *D. lowei* and *D. mauritiana* and https://www.diark.org/diark/species_list/Drosophila for *D. malerkotliana pallens*. We also used draft genomes of the three *Zaprionus* species (*Z. africanus*, *Z. indianus,* and *Z. gabonicus*), for which we provide RepeatMasker outputs, but the genome assemblies are not yet available (Haudry, pers.com.). On each assembly, RepeatMasker was run using the complete Repbase library to detect all elements in each genome and their closest match, using the *Drosophila* model. We then used the program OneCodeToFindThemAll [57] to parse RepeatMasker outputs, identify copies with respect to the 80–80-80 rule (using the “strict” option) and export sequences in FASTA format. From each set containing all 80–80-80 copies of the genome, a manually curated Repbase library, 111 Jockey families of *Drosophila* containing sequences of transcriptase reverses [Additional file 4: Data S1] was blasted to select Jockey copies with RT, with the option of evalue <1e-5 and a percentage of identity > 80%. Finally, a custom script was run to select the sequences corresponding to the three best hits (based on the bitscore criteria) per Jockey family in FASTA format.

All Jockey sequences extracted from the genomes were submitted to the NCBI’s Conserved Domains Database [58] for identification of the RT domain region, and sequences that presented a minimum extension of 300 nt for this domain were selected for the analyses. A list of the species in whose genomes Jockey sequences that met the search criteria were found is given [Additional file 1: Table S3].

### Phylogenetic analyses

Phylogenetic analyses were performed using the RT gene sequences, one of the most conserved regions of TEs that is, as a rule, used for phylogenetic analyses. The alignment was performed with MAFFT [59] and trimmed visually. Poorly aligned regions were removed using trimALL version 1.3 [60]. Then, sequences that aligned poorly or ≤ 200 bp of the filtered alignment were also removed. For the phylogenetic reconstructions, Bayesian inference (BI) was performed using BEAST v16.1 [61] with an a posteriori phylogenetic support test, using sampling of 100,000 trees and a burn-in of 10%. The times of divergence of the Jockey sequences belonging to the same family from the most recent common ancestor (MRCA) sequence were estimated using the Bayesian approach and BEAST v16.1, with a neutral nucleotide substitution rate of r = 0.016/site/My [62] for the calibration of the phylogenetic tree. Moreover, pairwise (p) distances between the *Jockey* sequences were estimated to classify sets of sequences belonging to the same family following the 80–80-80 criterion of Wicker et al. [15] using MEGA7 [63]. An evolutionary lineage was defined as a group that branched off early in the phylogeny and had an a posteriori probability ≥0.7. Within each lineage, the later clades, which include sequences with a divergence (p-distance) < 20% (similarity of ≥80%) and support of ≥0.7 or single sequences, constitute the families. Two clades of the same lineage constitute different families when the mean divergence between them is > 20%, which corresponds to a similarity < 80%.

The phylogeny of the drosophilid species involved in this study was reconstructed based on 48 sequences of the nuclear gene *Amyrel*, which was obtained from GenBank for most of the species (Additional file 1: Table S4). For *Z. gabonicus* and *Z. africanus*, the sequences were retrieved from the genomes using the *Z. indianus* sequence (EF458322.1) as a query. Bayesian inference was performed with BEAST v16.1 [61] using General Time Reversible (plus Gamma distribution and invariable sites) as the substitution model and an a posteriori phylogenetic support test, using sampling of 100,000 trees and a burn-in of 10%. The evolutionary model of substitution that best fit the data was determined by the Find Best DNA Model in MEGA7 [63].

### Inference of HTT

The inference of HTT was initially carried out by the classical criteria: sequence similarity and phylogenetic incongruence. When the necessary data were available, these inferences were validated by the application of VHICA (vertical and horizontal inheritance consistence analysis) methodology, which provides statistical support for corroboration of the inferences of HTT [18]. The method is based on discrepancies between the rate of evolution at synonymous sites (dS) and the ENC between pairs of TE sequences and vertically transferred orthologous genes. ENC is used to estimate selection due to codon usage bias (CUB) at synonymous sites. The use of dS and CUB together provides robustness in the HTT inferences because being dS and CUB correlated, a low dS does not necessarily indicates HTT if associated with a high CUB, but low dS associated with low CUB is inconsistent with VT [18]. Statistical support for the HTT inferences is given by a linear regression between the distribution of ENC and dS values (with Bonferroni correction, *P* < 0.01). For VHICA application, up to 29 single copy orthologous genes of each analysed species were used (Additional file 1: Table S5), with the exception for species involved in HTT with *D. lacertosa*, *D. albomicans* and *S. lebanonensis*, in which it was possible to extract only 19, 16 and 12 genes with good sequence quality, respectively, due to poor genome quality. The sequences of these genes were aligned using MAFFT [59] and later concatenated to reconstruct a phylogenetic tree of the host species. Additionally, the tree was inferred by maximum likelihood (ML), as described in Simão et al. [9].

### Gene and TE sequences used in the VHICA analysis

For the candidates of HTT among species (Fig. 1), we performed de novo gene prediction using Augustus [64], with “fly” as the training species, except for *D. erecta*, for which annotated CDSs were retrieved from the file “Drosophila_erecta.dere_caf1.cds.all.fa” available from the NCBI (https://www.ncbi.nlm.nih.gov/). A custom nucleotide database was created for each species that contained all the predicted/annotated (for *D. erecta*) coding sequences using makeblastdb and then used for a blastn search against the sequences of 50 single copy *D. melanogaster* genes from a set of 50 *Drosophila* single copy orthologous genes [18], using the following command: blastn -db $species.codingseq -query dmel_vhica_genes.fas -outfmt 6| sort -k1,1 -k12,12nr -k11,11n | sort -u -k1,1 --merge >best_single_hits.blastn. Probably due to variations in sequencing quality and genomic assemblies, as well to sequence divergence, the maximum number of genes found in all genomes, with sufficient quality and length to be used in the analysis was 29. For the TE dS estimation we selected the best copy of each TE family extracted from the genomes, regarding its length and sequence integrity.

## Supplementary information

**Additional file 1: Table S1.** Genome accessions for 48 species analysed. **Table S2.** Species of the genus *Drosophila* and *Zaprionus* analysed in this study, its hierarchical divisions and geographical origin. **Table S3**. Number of Jockey families and classification after phylogenetic analyses in 41 species of Drosophilidae. **Table S4.** Orthologous single copy genes vertically-transmitted genes used for each species used for the VHICA analyses. **Table S5.** Accession of Amyrel sequences used for reconstructing the species phylogeny.

**Additional file 2: Table S1.** Pairwise distances (p-distances) between 72 Jockey families. **Table S2**. Pairwise distances (p-distances) and similarity (1-p*100) within 72 Jockey families.

**Additional file 3: Figure S1.** Identification of Jockey elements horizontal transfer. Consistency graphical representation and dS-ENC graphs obtained from the comparison between species in Jockey-F45 representing inferences of HTT between *D. yakuba* and *D. simulans*. For details of the graphs refer to the legend of Fig. 2. **Figure S2.** Identification of Jockey elements horizontal transfer. Consistency graphical representation and dS-ENC graphs obtained from the comparison between species in Jockey-F6 (**a**), Jockey-F13 (**b**) and Jockey-F23 (**c**) representing inferences of HTT and VT. For details of the graphs refer to the legend of Fig. 2. There are signals of HTT between *D. takahashii* and *D. elegans* in both Jockey-F13 and Jockey-F23. **Figure S3.** Identification of Jockey elements horizontal transfer. Consistency graphical representation and dS-ENC graphs obtained from the comparison between species in Jockey-F39 (**a**), Jockey-F62 (**b**) and Jockey-F67 (**c**) representing inferences of HTT and VT. For details of the graphs refer to the legend of Fig. 2. There are signals of HTT between *D. takahashii* and *Z. africanus* (Jockey-F39) and *S. lebanonensis* and *D. obscura* (Jockey-F62).

**Additional file 4: Data S1**. Sequences of the transcriptase reverse gene from 111 families of Jockey clade downloaded from Repbase database [16] that occurs in species of the genus *Drosophila*.

## Data Availability

All data generated or analysed during this study are included in this published article and its supplementary information files. Jockey sequences dataset is available from the corresponding author on reasonable request.

## Abbreviations

CUB: Codon Usage Bias
dS: Synonymous sites
ENC: Preferential use of codons
HT: Horizontal transfer
HTT: Horizontal transfer of transposable elements
LINE: Long interspersed nuclear element
MRCA: Most Recent Common Ancestral
Mya: millions of years ago
TEs: Transposable elements
VHICA: Vertical and Horizontal Inheritance Consistence Analysis
VT: Vertical transfer
