# Supplementary material for: High frequency of horizontal transfer in Jockey families (LINE order) of drosophilids
Source: Mob DNA. 2019 Nov 4;10:43. doi: 10.1186/s13100-019-0184-1 (PMC6829985; doi:10.1186/s13100-019-0184-1)
Supplement: Supplementary file 3 — Additional file 3: Figure S1. Identification of Jockey elements horizontal transfer. Consistency graphical representation and dS-ENC graphs obtained from the comparison between species in Jockey-F45 representing inferences of HTT between D. yakuba and D. simulans. For details of the graphs refer to the legend of Fig. 2. Figure S2. Identification of Jockey elements horizontal transfer. Consistency graphical representation and dS-ENC graphs obtained from the comparison between species in Jockey-F6 (a), Jockey-F13 (b) and Jockey-F23 (c) representing inferences of HTT and VT. For details of the graphs refer to the legend of Fig. 2. There are signals of HTT between D. takahashii and D. elegans in both Jockey-F13 and Jockey-F23. Figure S3. Identification of Jockey elements horizontal transfer. Consistency graphical representation and dS-ENC graphs obtained from the comparison between species in Jockey-F39 (a), Jockey-F62 (b) and Jockey-F67 (c) representing inferences of HTT and VT. For details of the graphs refer to the legend of Fig. 2. There are signals of HTT between D. takahashii and Z. africanus (Jockey-F39) and S. lebanonensis and D. obscura (Jockey-F62). [file 13100_2019_184_MOESM3_ESM.pdf]

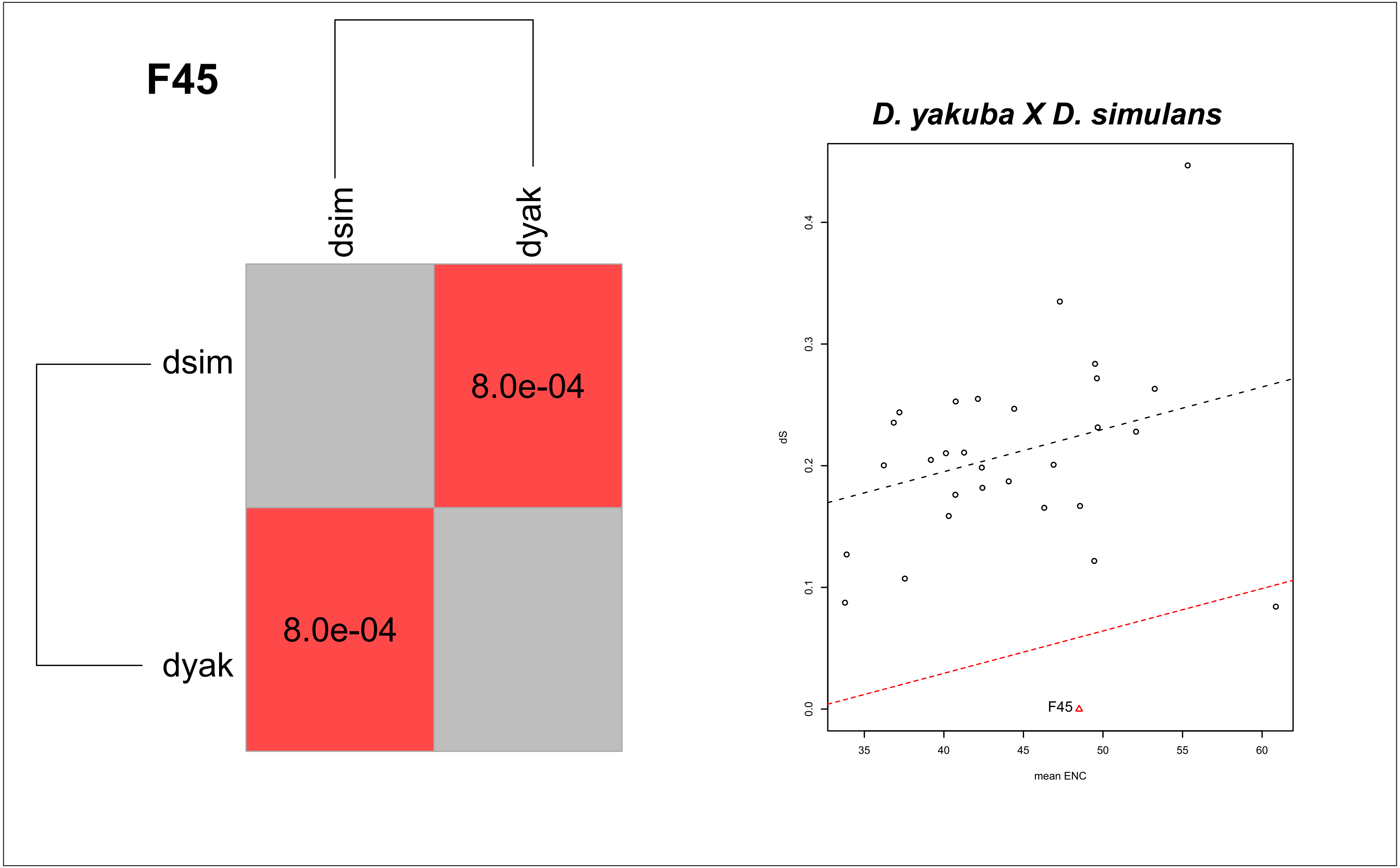

F45
dsim
dyak
dsim
dyak
8.0e-04
8.0e-04
D. yakuba X D. simulans
dS
0.0
0.1
0.2
0.3
0.4
35
40
45
50
55
60
mean ENC
F45

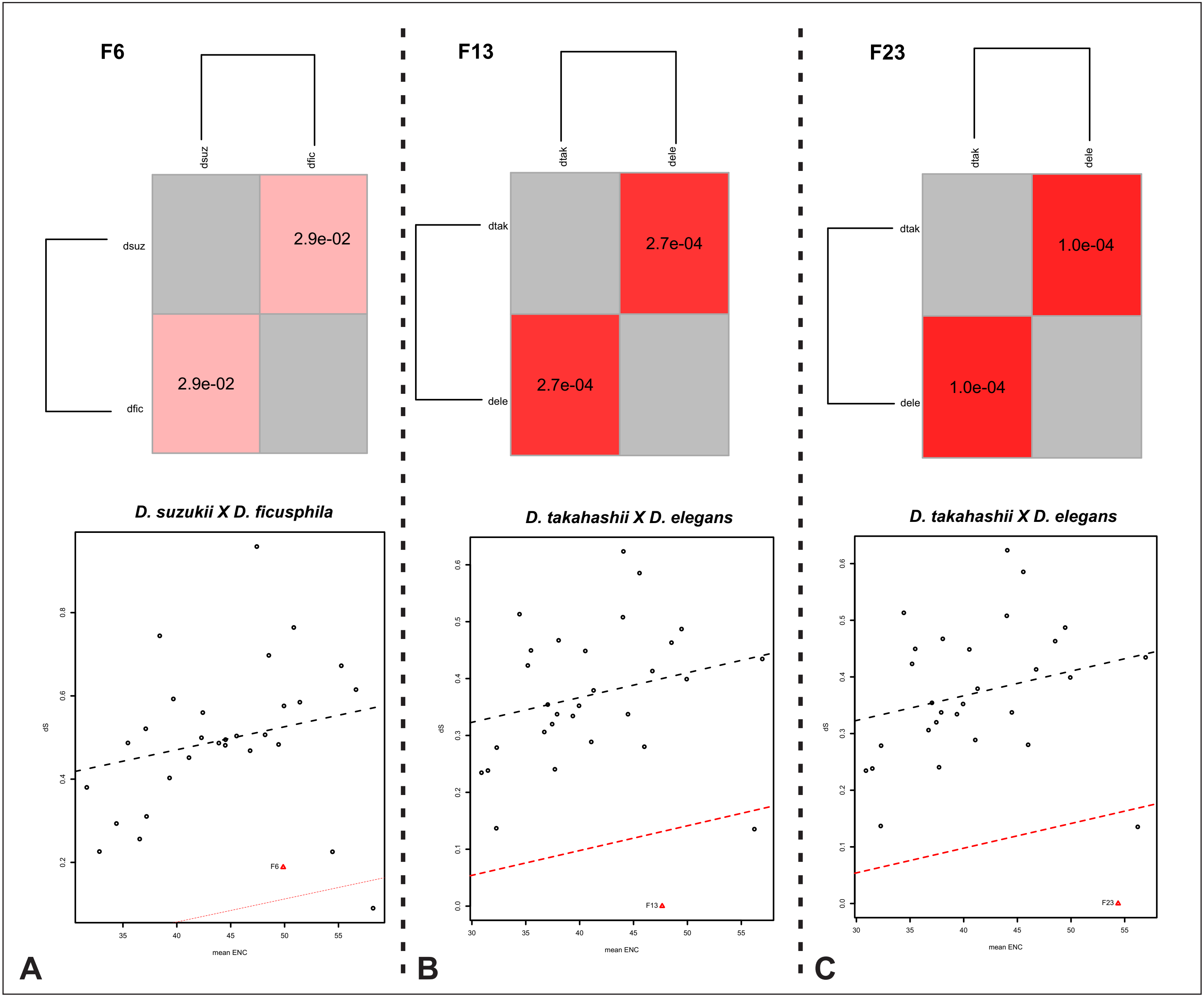

F6
F13
F23
dsuz
dfic
2.9e-02
2.9e-02
dtak
dele
2.7e-04
2.7e-04
1.0e-04
1.0e-04
D. suzukii X D. ficusphila
D. takahashii X D. elegans
D. takahashii X D. elegans
dS
mean ENC
F6
F13
F23
A
B
C

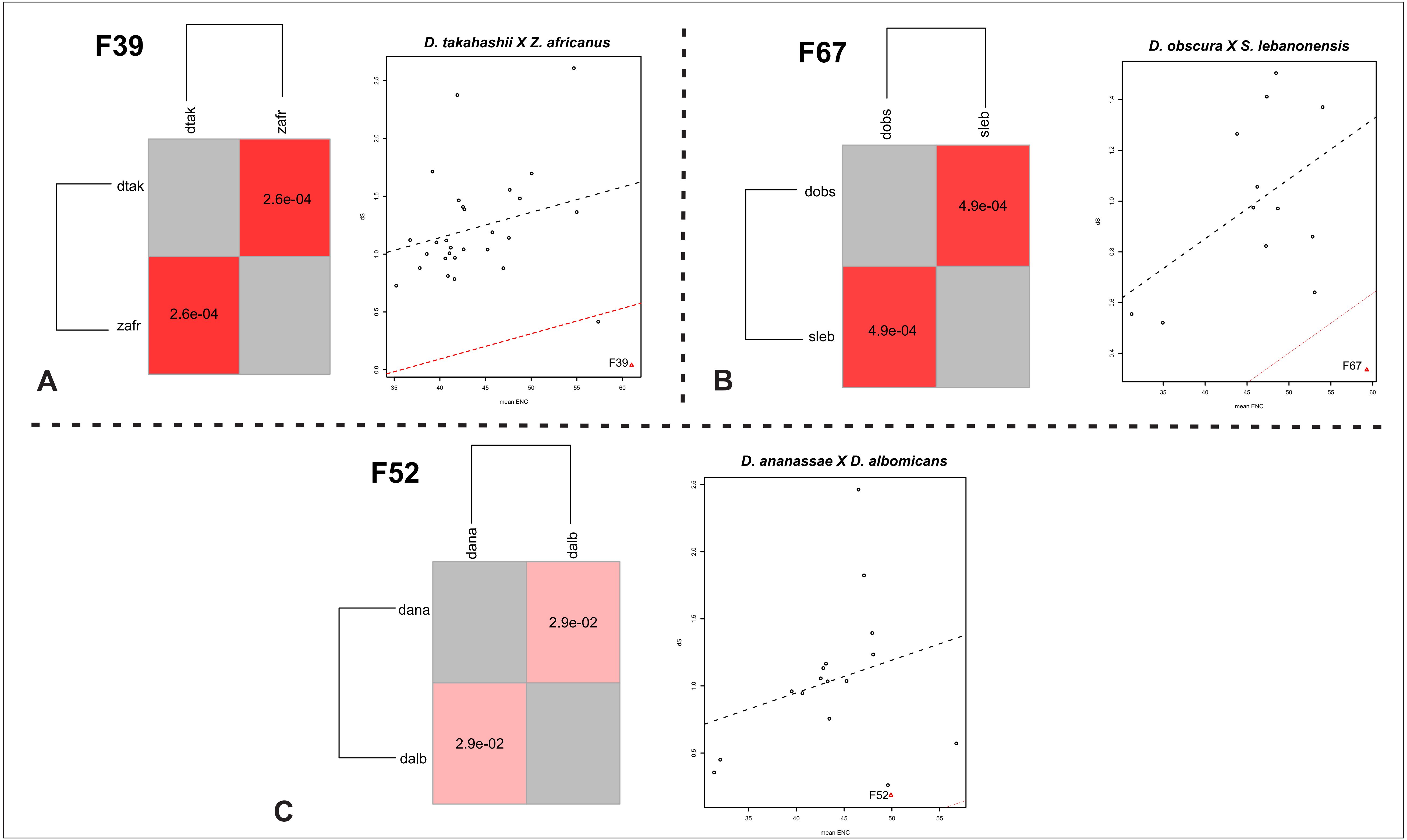
F39
dtak
zafr
2.6e-04
2.6e-04
A
D. takahashii X Z. africanus
dS
mean ENC
F39
F67
dobs
sleb
4.9e-04
4.9e-04
B
D. obscura X S. lebanonensis
dS
mean ENC
F67
F52
dana
dalb
2.9e-02
2.9e-02
C
D. ananassae X D. albomicans
dS
mean ENC
F52
